# Cytoskeletal and Cytoskeleton-Associated Proteins: Key Regulators of Cancer Stem Cell Properties

**DOI:** 10.3390/ph15111369

**Published:** 2022-11-08

**Authors:** Yuqiang Li, Dan Wang, Heming Ge, Cenap Güngör, Xuejun Gong, Yongheng Chen

**Affiliations:** 1 Department of General Surgery, Xiangya Hospital, Central South University, Changsha 410008, China; 2 National Clinical Research Center for Geriatric Disorders, Xiangya Hospital, Central South University, Changsha 410008, China; 3 NHC Key Laboratory of Cancer Proteomics, Laboratory of Structural Biology, Xiangya Hospital, Central South University, Changsha 410008, China; 4 Department of General Visceral and Thoracic Surgery, University Medical Center Hamburg-Eppendorf, 20246 Hamburg, Germany

**Keywords:** cytoskeletal, cytoskeleton-associated proteins, cancer stem cells, recurrence, metastasis, drug resistance

## Abstract

Cancer stem cells (CSCs) are a subpopulation of cancer cells possessing stemness characteristics that are closely associated with tumor proliferation, recurrence and resistance to therapy. Recent studies have shown that different cytoskeletal components and remodeling processes have a profound impact on the behavior of CSCs. In this review, we outline the different cytoskeletal components regulating the properties of CSCs and discuss current and ongoing therapeutic strategies targeting the cytoskeleton. Given the many challenges currently faced in targeted cancer therapy, a deeper comprehension of the molecular events involved in the interaction of the cytoskeleton and CSCs will help us identify more effective therapeutic strategies to eliminate CSCs and ultimately improve patient survival.

## 1. Introduction

A steady stream of research has led to a degree of understanding of the tumorigenesis and growth of primary tumors and the development of complex and effective treatments that can significantly prolong patient survival. However, an inescapable problem is that tumor recurrence and metastasis remain a major cause of high mortality in patients with cancer, even after radical surgery combined with adjuvant therapy [1]. Moreover, chemotherapy resistance is also a troubling and intractable problem in the course of tumor treatment [1]. However, the effective understanding of both phenomena is still very limited. Theories related to cancer stem cells (CSCs), as a rising star in tumor research, seem to help understand the abovementioned problems to a certain extent. CSC theory suggests that CSCs can drive tumor growth, promote tumor progression, and initiate mechanisms related to distant metastasis and drug resistance, features that ultimately lead to dismal clinical outcomes [2,3,4]. Therefore, the eradication of this specific group seems to be a priority after recognizing that CSCs with these characteristics may be the culprits. Before addressing this issue, a comprehensive understanding of the biological drive, state regulation and maintenance of CSCs is necessary.

Similar to the case of normal stem cells, CSCs are thought to present in a niche [5]. The niche of CSCs is their specific survival microenvironment, which regulates the fate of CSCs through secreted factors and cell–cell contacts [5]. Niches are three-dimensional structures composed of extracellular matrix components, signaling molecules, and other cells [6]. Through the mechanical interaction of their niche, cells are mechanically loaded, resulting in the possible deformation of the cell membrane, cytoskeleton, and nucleus, thus triggering the secretion of relevant signaling molecules into the niche [5,6]. These signals and secretory factors in turn regulate the metabolism, morphology, and mechano-sensitivity of secretory cells [6]. One study reported that the cytoskeleton is closely related to the activity of CSCs [7]. The eukaryotic cytoskeleton, which is made up of microfilaments, intermediate filaments, and microtubules, is a dynamic and intricate three-dimensional network that exists inside the cytoplasm [8]. The main roles of the cytoskeleton include mechanical support, cell shape regulation, the facilitation of cell migration, and intracellular transport [8,9]. It can also provide locations for the localization and binding of signaling molecules as scaffolds for signaling cascades [8,9]. The dysregulation of the cytoskeleton is closely related to a variety of diseases, especially cancer [10]. Different cytoskeletal components and remodeling processes have profound effects on the behavior of CSCs [11]. Moreover, the cytoskeleton can play a role in regulating cellular bioenergetics in CSCs by dynamically controlling the mitochondrial structure and function of CSCs, in addition to affecting the niche of CSCs [12].

It is evident that understanding the influence of the cytoskeleton and related proteins on various biological behaviors of CSCs will help to further solve the clinical treatment challenges of cancer. Therefore, we outline existing research that supports the significance of cytoskeletal components in controlling the structure, bioenergetics, and function of CSCs. A greater understanding of the behavior and regulatory factors of CSCs will help with the creation of innovative treatments for metastatic, drug-resistant malignancies.

## 2. Cancer Stem Cells

In the early 1990s, CSCs were discovered in leukemia and were isolated by recognizing the expression of their characteristic surface markers, CD34^+^CD38^−^ [13,14]. Subsequently, CSCs expressing different surface markers were identified in a large number of solid tumors, such as CD133^+^CXCR4^+^ CSCs in pancreatic cancer and CD44^+^CD24^−^ CSCs in breast cancer [15,16]. It was shown that these special cells are also part of the tumor body [17]. CSCs have a strong capacity for self-renewal, that is, the process of generating at least one daughter cell that retains stem cell characteristics by symmetric or asymmetric division [18]. The expansion of CSCs in a symmetrical division leads to unrestricted cell growth, which directly leads to tumor formation [19,20]. CSCs, like regular stem cells, are controlled by the Wnt/β-catenin, Sonic Hedgehog (Hh), and Notch pathways responsible for self-renewal [21,22,23]. Understanding the regulation of CSCs’ self-renewal may provide more options for cancer treatment. Another great feature of CSCs is their capacity to differentiate into various cell types [24]. Under normal circumstances, multiple signaling pathways stably regulate these two important properties of stem cells to form a balance that is conducive to normal proliferation and differentiation [25]. However, when this regulatory balance is disrupted, uncontrolled CSCs grow and migrate in a frantic manner, ultimately leading to tumor progression and metastasis [26].

CSCs are located in niches, specialized anatomical areas within the tumor microenvironment [27]. These unique niches contribute to the maintenance of the aforementioned characteristics of CSCs and promote their phenotypic flexibility while shielding them from the immune system [23,28]. Aberrant tumor proliferation and vascular rarefaction lead to a tumor microenvironment characterized by hypoxia, acidity, and malnutrition [29]. Therefore, CSCs must effectively adapt their cellular bioenergetics to cope with these adverse conditions [30]. An in-depth study revealed that CSCs prefer mitochondrial oxidative metabolism [31]. Cancer cells carry out aerobic glycolytic metabolism, while CSCs mainly rely on oxidative phosphorylation (OXPHOS) [32,33,34]. Subpopulations of cancer cells switch between glycolysis and OXPHOS to meet the energy demands of survival, also embodying metabolic plasticity [35]. Compared with general tumor cells, mitochondrial mass and membrane potential were found to be increased in CSCs, reflecting an enhanced mitochondrial function and increased oxygen consumption rate [36,37,38,39,40]. Additionally, a high mitochondrial mass suggests a stem cell phenotype that is linked to a potential for metastasis and resistance to DNA damage [41]. Furthermore, CSCs are considered to be an important contributor to chemoresistance due to their well-defined quiescent phenotype, endothelial–mesenchymal transformation (EMT), multidrug resistance (MDR), and resistance to DNA damage-induced apoptosis [42,43,44,45].

## 3. Cytoskeleton of the Cell

The cytoskeleton is mainly composed of three structures: microfilaments made of G-actin and F-actin, microtubules made of α and β-tubulin, and intermediate filaments (IFs) made of different keratins and vimentin [8,46]. Any changes, including those of the cellular structure and the rearrangement and relocation of organelles, may lead to changes in cellular metabolism that enhance cell migration and invasion characteristics [9]. Actin and microtubules in the cytoskeleton play supportive and key regulatory roles in these important cellular processes [47]. The unfolding of the actin network is essential to the majority of cellular processes [48]. The ability of actin to freely switch between polymeric F and monomeric G actin forms confers the fast remodeling of the actin cytoskeleton in response to internal and external stimuli [49]. Moreover, this cytoskeletal remodeling plays a crucial role in cellular integrity, motility, and membrane trafficking [50]. Actin can synthesize slowly growing pointy ends and quickly growing barbs through self-polymerization in vitro, and intracellular polymerization is tightly regulated by actin nucleation and actin-severing proteins [51]. Moreover, actin filaments can generate various pseudopodia that cells might exploit to investigate the extracellular environment during invasion and metastasis [52]. Actin reorganization also occurs during mitochondrial fission [53]. Mitochondria can travel via dendrites and axons along actin filaments [12]. During the actin breakdown phase, fragmented mitochondria rapidly fuse, accelerating mitochondrial integrity repair and maintaining mitochondrial homeostasis [54]. In addition, F-actin cages around dysfunctional mitochondria are triggered to assemble, preventing the proliferation of damaged mitochondria [55]. In conclusion, the actin cytoskeleton is critical for the spatial domain transport, dynamics and quality control of mitochondria. Actin-nucleating agents that stimulate the formation of actin filaments are essential for actin activity. Known actin-nucleating proteins include formins, tandem WASP homology 2 (WH2) nucleators, and the Arp2/3 complex [56]. The ability to polymerize and depolymerize is critical to actin, and these processes are primarily regulated in space and time by the actin-binding protein (ABP) family [57]. In cancer cells, the homeostasis between G and F actin and their association with ABPs is frequently altered, leading to dysregulation [58]. ABPs are categorized as monomer-binding proteins, cross-linking and binding proteins, end-capping and severing proteins, anchoring proteins, signaling proteins, and stabilizing proteins based on their roles [59].

### 3.1. Monomer-Binding Proteins

Monomer-binding proteins mainly include profilin, twinfilin, and thymosin β4 [60]. Four different types of profilin (PFN) exist (PFN1-4), with PFN1 being extensively expressed in all tissues [61]. PFN catalyzes the exchange of ADP with ATP on G-actin monomers, which is essential for actin polymerization [62]. PFN modulates membrane protrusion by binding to N-WASP and VASP, which also increases the intracellular PFN concentration and enhances the elimination of stinger-terminated actin monomers, resulting in depolymerization [63]. The phosphorylation/dephosphorylation of serine 3 is a major regulator of PFN activity. In addition, in combination with PIP2 and cortactin, intracellular pH also modulates the activity of PFN itself [64]. Twinfilin (TWF), an actin monomer sequestering protein, exists in two isoforms of TWF-1 and -2 in the human body [65]. TWF is mainly found in lamellar pseudopods, localized in subcellular regions with a high actin turnover. By binding to ADP-G actin, TWF prevents G-actin from being added to actin filaments [66]. Thus, TWF mainly regulates actin polymerization/depolymerization by blocking nucleotide exchange on actin monomers [67]. Furthermore, TWF is involved in cell migration and the EMT, and it controls the cell cycle by affecting the mTOR pathway [68,69]. Its synthesis is regulated by Cdc42-downstream signaling and Rho GTPases Rac1 [65]. Thymosin β4 (Tβ4) is involved in cytoskeletal reorganization by buffering intracellular G-actin concentrations [70]. It is itself a significant G-actin sequesterer that activates Cdc42 and Rac by activating various signaling pathways [71,72]. Moreover, many studies have suggested that Tβ4 can activate the hypoxia-inducible factor 1 (HIF-1) gene and participate in processes such as the EMT and angiogenesis through the AKT and Notch/NF-κB pathways [73,74,75].

### 3.2. Cross-Linking and Bundling Proteins

Members of the cross-linking and bundling protein family include fascin, filamins, spectrins and alpha-actinin [76]. Fascin, required for actin binding and bundling, is present in tissues as three isoforms (fascin-1, -2, and -3) [77]. The special beta trefoil of fascin forms actin-binding sites that bind dozens of parallel actin filaments together to form tight, stiff filamentous pseudopods [78]. These actin bundles act as proprietary channels that deliver signaling molecules from the cell core to the cell leading edge [79]. The Rho family of GTPases is a small (~21 kDa) family of signaling G proteins, of which members RhoA and Rac1 act upstream of fascin via protein kinase C (cPKC) to regulate actin binding [80]. F-actin cross-linking by fascin-1 involves the *N*-terminal and *C*-terminal domains of fascin-1, and a major mechanism that inhibits the actin-bundling activity of fascin-1 is the phosphorylation of an N-terminal motif (S39 in human fascin-1) by conventional isoforms of protein kinase C (cPKC) [81]. Rac1 and RhoA inhibit actin binding by promoting the cPKC phosphorylation of S39 [82]. Of the three isoforms of filamin (FLN), FLNA and FLNB are widespread while FLNC is confined to cardiac and skeletal muscle [83]. One of the main roles of FLN is to connect actin filaments to the cell membrane [84]. FLN is a homodimeric protein found in stress fibers, lamellar pseudopods and filamentous pseudopods [48]. The N-terminal domain is an actin-binding region featuring F-actin-, α-actin-, β-spectrin-, and fibrin-binding sites [85]. The *C*-terminus is a repeating rod region through which protein dimerization occurs in a tail-to-tail manner [86]. Functionally, in addition to helping actin form orthogonal branches, FLNA is involved in linking many receptors related to cell signaling and the cell cycle [87]. FLNA is mainly regulated by the phosphorylation of residue S2152 [88]. Spectrins construct hexagonal lattices beneath the plasma membrane to keep the membrane cytoskeletal network stable [89]. The α- and β-spectrin genes, which are widespread in cells, encode the two isoforms of spectrin [90]. The α and β subunits are arranged head-to-head to form an antiparallel tetramer that constitute the platform for the binding of channel proteins, receptors, and transporters [91]. On the one hand, spectrin participates in cell migration through actin-dependent and non-actin-dependent mechanisms, and it can bind to calcium or calmodulin to participate in cell proliferation [92]. This is due to the fact that at normal calcium levels, spectrin proteins are found along the edges of cells and diffuse throughout the cell as calcium concentrations increase [93]. Spectrin binds to calmodulin-dependent protein kinase II (CaMKII), which activates the PI3K/Akt signaling pathway to promote proliferation [94]. Additionally, spectrin is involved in hypoxia-induced, angiogenesis-mediated cytoskeletal remodeling, a process regulated by c-Jun *N*-terminal kinase (JNK) signaling [95]. The α actinin (ACTN) that belongs to the spectrin superfamily is present in all cells [96]. ACTN exists in four forms (ACTN 1-4) in the human body and is crucial for the formation and stabilization of stress fibers [96,97]. ACTN links the cytoskeleton with transmembrane proteins, stabilizes the cell structure, and provides a scaffold for the integration of signaling molecules into specific sites [98]. The PIP3 produced by PI3K activation damages the interaction of ACTN with actin and integrins, thereby impairing the structure of focal adhesion and promoting cytoskeletal remodeling [99].

### 3.3. Anchoring Proteins

Anchoring proteins mainly include the ezrin–radixin–moesin (ERM) family and merlin [100]. Ezrin is mainly expressed by the vil2 gene in epithelial cells [101]. Its C and N domains interact with the integral membrane proteins in the actin cytoskeleton and the plasma membrane, respectively [102]. This allows ezrin to form connections between the actin cytoskeleton and the plasma membrane and to respond to extracellular signals [103]. Ezrin is also closely associated with multiple signaling pathways, including Rho, PI3K, AKT, and MAPK [104]. Moesin, another member of the ERM family, binds to actin via the C-terminus, thereby attaching actin to the plasma membrane [105]. Moesin, localized in filopodia and microvilli, is involved in the EMT, cell adhesion, and membrane fold formation [106,107]. In addition, moesin affects cell division and spindle–actin communication by binding to microtubules [108]. Radixin, encoded by chromosome 11, has a central α-structural domain with an F-actin-binding site at the *C*-terminus [109]. Radixin is essential for cytoskeletal organization, as well as cell motility and adhesion, as it cross-links to actin on the cell surface [110]. Merlin, encoded by chromosome 22, has two isoforms: isoform 1 and isoform 2 [111]. Lacking a conserved actin-binding site, the N-terminus of merlin is the actin-binding domain [112]. Merlin plays an important role in the intracellular effectors that control cell proliferation and adhesion, as well as linking F-actin and transmembrane receptors [112].

### 3.4. Capping and Severing Proteins

Gelsolin is a ubiquitous capping and severing protein that interacts with G- and F-type actin to regulate actin polymerization through severing, capping, and nucleation [113]. The gene encoding it produces two isoforms that are localized in the plasma and cytoplasm [114]. Gelsolin directly or indirectly alters lipid signaling by binding to kinases and lipases [115]. Cofilins are evolutionarily conserved capping and severing proteins [116]. Cofilin has binding sites for both F-actin and monomeric G-actin. The binding of cofilin to actin filaments alters the orientation of the subunit, which leads to filament severance, producing barbed ends, the preferred site for Arp2/3 binding [117]. The regulation of cofilin mainly depends on the phosphorylation of the LIMK/TESK kinase at ser-3 or pH, PIP2, or cysteine oxidation [118,119]. Villin is a significant part of the brush border cytoskeleton in differentiated epithelial tissue, where it binds, caps, severs and bundles actin filaments [120]. Villin has three actin-binding sites, two of which retain calcium-dependent activity at the core [121]. Villin maintains an autoinhibitory conformation at normal physiological calcium concentrations, but its structural conformation changes and binds to actin with increasing intracellular calcium concentrations [122]. The phosphorylation of tyrosine residues within the core of villin promotes actin severing and binding in multiple modalities, thereby increasing cytoskeletal fluidity and affecting its mechanical properties, ultimately enhancing cell movement [123]. A study showed that the binding of villin to F-actin is regulated by calcium concentration, PIP2 or tropomyosin [124].

### 3.5. Stabilizing Protein and Signaling Protein

**Figure 1:**
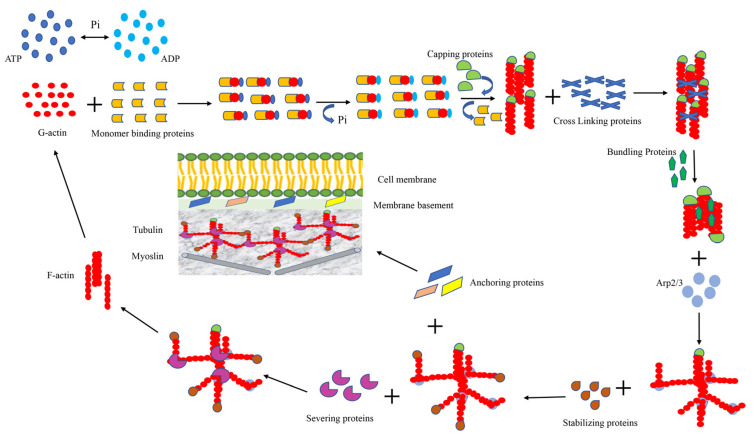
Actin-binding proteins. This figure shows the proteins involved in actin binding, capping, cross-linking, bundling, severing and anchoring.

Tropomodulins are stabilizing proteins that wrap around the growing end of actin to prevent the dissociation or addition of G-actin [125]. In addition, tropomodulins can regulate actin dynamics by acting as actin-nucleating agents [125]. Furthermore, tropomodulins regulate actin filament assembly, stability and length via capping [126]. The nucleotide concentration of actin affects tropomodulins’ affinity for G-actin monomers [127]. Ena/VASP is a signaling protein that is essential in the formation and elongation of filamentous pseudopods [128]. The C-terminus of this signaling protein has binding sites for G and F actin, and the protein itself promotes tetramerization, which is significant for actin extension [129]. Ena/VASP proteins are conserved regulators of actin dynamics and play important roles in a variety of physiological processes including morphogenesis, axon guidance, endothelial barrier function, and cancer cell invasion and metastasis [130]. The anti-capping model of Ena/VASP function appears to be the simplest explanation for many of the known cellular biological and biochemical properties of this protein family. Another biochemical property of Ena/VASP proteins is their ability to nucleate actin filaments in vitro, but the importance of this effect in vivo remains to be confirmed [130]. The interaction of Ena/VASP with actin and other proteins can be affected by the phosphorylation of PKA and the dephosphorylation of protein phosphatases [131] (Figure 1).

### 3.6. Microtubule-Associated Proteins

Microtubules are one of the fundamental constituents of the cytoskeleton and are composed of α- and β-tubulin heterodimers bound together [132]. Microtubules play critical roles in cell morphology, cell division, vesicle transport and cell signaling [132]. Microtubule-associated proteins (MAPs) bind to microtubules, connect them to other organelles, bind them, and transport related substances [133]. Tau is a common MAP that manages microtubule polymerization and stability to govern microtubule protein dynamics [134]. Excessive phosphorylation leads to a decreased affinity between Tau and microtubules, which alters post-translational modifications and destabilizes the cytoskeleton, ultimately resulting in a diminished EMT and invasiveness [135]. Microtubule-associated protein 2 (MAP2) stabilizes microtubule growth by cross-linking microtubules to intermediate filaments, leading to microtubule stiffness activation [136]. Katanin is a heterodimeric protein composed of katanin P60 and katanin P80 subunits that exerts its microtubule-cutting function by deploying ATP hydrolysis to extract microtubulin dimers at the lattice and break down the polymer [137]. In addition, angiotensin II receptor-interacting protein 3 (ATIP3), one of the structural MAPs localized along the microtubule lattice, is an effective microtubule stabilizer that binds to end-binding proteins (such as EB1) in the cytoplasmic lysate, thereby attenuating microtubule dynamics [138]. The four abovementioned MAPs belong to microtubule lattice-binding proteins, which are localized along the length of microtubules.

**Figure 2:**
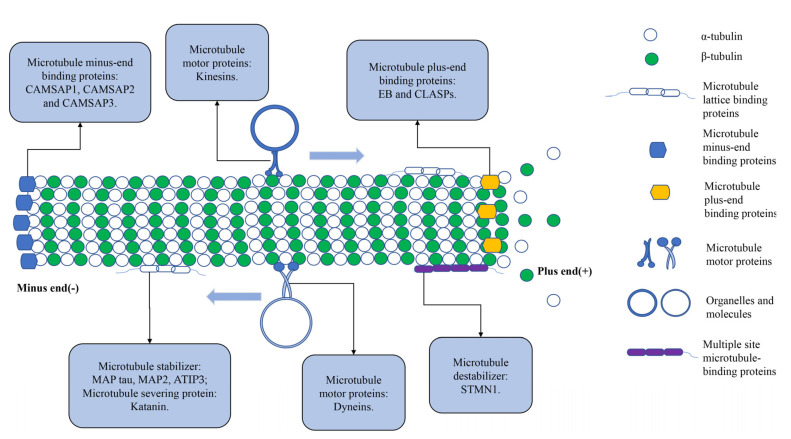
Schematic representation of microtubule−associated protein (MAP) binding to microtubules at different sites. The main proteins include microtubule crystal−binding proteins (MAP tau, MAP2, ATIP3, and katanin), microtubule movement proteins in which kinesins carry organelles or molecules to the plus−end of microtubules (anterograde transport); dyneins that transport cellular molecules to the minus−end of microtubules (retrograde transport); multisite microtubule−binding protein stathmin (STMN1); microtubule plus−end−binding proteins (EB and CLASPs); and microtubule minus−end-binding proteins (CAMSAP1, CAMSAP2 and CAMSAP3).

Microtubule motility proteins include kinesins and dyneins that transport molecules along microtubule tracks [139]. Kinesins consist of different isoforms of family proteins that are involved in individual cellular activities. Classical kinesin with an *N*-terminal motility domain and kinesin family member 14 (KIF14) with an intermediate motility domain deploy ATP hydrolysis to generate kinesin motility with mechanical force toward the plus-ends of growing microtubules [140,141]. Dyneins transport in the opposite direction to kinesins, moving toward the minus-end of microtubules and transporting intracellular cargo from the cell periphery to the center in a retrograde direction [142]. Kinesins and dyneins play important roles in different microtubule-dependent activities, intracellular vesicle transport, organelle transport, and mitotic spindle organization [143]. Stathmin (STMN1) is one of the most prominent microtubule destabilizers, reducing the length of microtubule polymers by indirectly binding microtubule protein subunits in a bent form, thus promoting depolymerization [144]. In addition, STMN1 induces microtubule instability by specifically interfering with the lateral binding of microtubule protein subunits at the microtubule ends, acting at both the plus- and minus-ends [145]. In addition, MAPs that preferentially contain polymerized microtubule plus-ends are referred to as plus-end tracking proteins (+TIPs) [146]. End-binding proteins (EBs) are typical +TIP protein types, including EB1, EB2 and EB3, which precisely bind to the plus-end of microtubules and associate with stable GTP caps for microtubule growth [147]. Cytoplasmic linker-associated proteins (CLASPs) are a conserved class of +TIPs proteins that contribute to microtubule stabilization [148]. Members of the calmodulin-regulated spectrin-associated protein (CAMSAP) family, consisting of CAMSAP1, CAMSAP2 and CAMSAP3, have recently been described as microtubule minus-end-binding proteins [149]. They may regulate the stability and localization of microtubule negative ends, thereby organizing non-centrosomal microtubule networks sufficient for cell division, migration and polarity [150] (Figure 2).

### 3.7. Other Components of the Cytoskeleton

The myosin superfamily includes myosins with different structures and functions encoded by dozens of genes [151]. The main role of myosin is to convert chemical signals into mechanical forces, a process that is achieved by its sliding along actin filaments involved in ATP hydrolysis [152]. A variety of important cellular functions such as intracellular signal transduction, cell migration and tumor suppression can be seen with traces of myosin involvement [153]. IFs are dynamic, nonpolar fibrillar structures highly concentrated in desmosomes and hemidesmosomes [154]. IFs share a common structure: an *N*-terminal head domain, a *C*-terminal tail domain, and a central rod domain [155]. IFs undergo dramatic structural changes upon the receipt of relevant signals, providing structural support and participating in the control of processes such as cellular proliferation and apoptosis [156]. The regulation of IFs’ organization mainly relies on the interaction of IFs with other proteins, the phosphorylation of some signaling pathways, or post-translational modifications [157]. According to different structures and localizations, intermediate filament proteins can be divided into: type I and type II-acidic and basic cytokeratins; type III-vimentin, glial fibrillary acidic protein, desmin, synchronization protein, and peripheral protein; type IV-neurofilament and α-internexin; type V-lamins; and type VI-synemin and nestin [158]. Keratin binds to integrins via plectin to stabilize hemidesmosomes, causing cell migration and adhesion to be stabilized [159]. Vimentin, which is abundantly expressed in normal mesenchymal cells, is primarily responsible for cellular integrity and stress tolerance [160].

## 4. Cytoskeleton and CSCs

CSCs are generally considered to be the main culprit for cancer metastasis and chemotherapy resistance. The different components of the cytoskeleton, remodeling processes, and interactions with CSCs enable CSCs to adapt to unique tumor microenvironments, thus maintaining cell stemness and migratory activity.

### 4.1. Actin and CSCs

Yes-associated protein (YAP) and transcriptional coactivator with PDZ-binding motif (TAZ) are important signaling molecules that regulate drug resistance and cancer stem cell biomechanics. [161]. YAP/TAZ proteins act as mechanosensors in response to physical stimuli involving the actin cytoskeleton [162]. YAP/TAZ proteins have been shown to play a two-sided role in the Wnt signaling pathway, which is critical for intercellular function and self-renewal capacity [163]. This is mainly because YAP/TAZ proteins are components of the β-catenin destruction complex that translocates to the nucleus upon the activation of the Wnt pathway [162]. An increased extracellular matrix (ECM) stiffness activates the YAP/TAZ-downstream Rho/ROCK pathway, which facilitates the survival of CSCs [164]. The activation of integrin and focal adhesion kinase (FAK) contributes to focal adhesion formation, leading to the activation of Rho-GTPase and stress fibrillogenesis. Meanwhile, focal adhesions require actin polymerization. These together lead to the repression of YAP/TAZ transcription factors, resulting in negative effects on CSCs [165]. In this case, myosin increases the tension on the actin network after a cell has spread, while F-actin reduces tension by dissociating to maintain tension balance [164].

### 4.2. Monomer-Binding Proteins and CSCs

PFN is essential for cell motility in vivo through the regulation of actin polymerization kinetics [166]. Cell migration and intercellular adhesion can be inhibited by reducing the expression of PFN in cancer cells [93]. In colorectal cancer, changes in the invasive, migratory and self-renewal abilities of HT29 CSCs were found to be consistent with the rise and fall of PFN2 expression levels. Furthermore, PFN2 directly regulates the expression of EMT markers (E-cadherin) and stemness markers (SOX2, CD133 and β-catenin) [167]. SOX2 is a transcription factor that is essential for the regenerative capacity of stem cells, as well as for the maintenance of pluripotency [168]. Thus, PFN2 plays an important role in the stemness and metastatic potential of CSCs by regulating related transcription factors. Additionally, the knockdown of PFN1 in breast cancer cells was shown to result in the diminished expression of CSC-related genes, further demonstrating the important regulatory role of PFN on CSC-related properties [169]. Tβ4 relies on the cytoskeletal organization of actin to exert a regulatory role on tumorigenicity and metastatic capacity in mouse fibrosarcoma cells [170]. Tβ4 is overexpressed in a variety of tumors and maintains the cell stemness of CSCs by increasing the EMT [171]. Because of its important role in promoting the tumorigenic properties of colorectal CSCs, Tβ4 may have important implications for therapeutic intervention in human colon cancer [172]. Moreover, the expression of Tβ4 is closely associated with the expression of the CSC marker CD133 in gastric and ovarian cancers, thus having an impact on tumor metastasis [173]. In pancreatic cancer, Tβ4 mainly regulates CSCs by activating the JNK pathway and promoting the expression of pro-inflammatory cytokines, thereby promoting cancer progression [174]. This may be due to the fact that Tβ4 first enhances the bone morphogenetic protein (BMP) pathway, which activates JNK through the TAB1 and TAK1 complex [175,176]. Of course, further studies are needed to elucidate the exact pathway of Tβ4-induced JNK activation. Additionally, the increased expression of Tβ4 promotes the migration and metastasis of CSCs, mainly through the activation of Rac and the elevation of the IQGAP1/ILK complex [177]. TWF, a conserved actin-binding protein, is also a prime candidate target for the downregulation list of miR-206 [178]. It has been reported that hsa-miR-206 attenuates the stemness and metastatic ability of breast CSCs by reducing their self-renewal and invasive ability. TWF1 could rescue the invasive phenotype of miR-206 by enhancing the activity of the mesenchymal lineage transcription factor-megakaryocytic leukemia 1 (MKL1) and actin cytoskeleton dynamics [178]. On the other hand, a systemic RNA interference screening study revealed a strong association of TWF1 with chemosensitivity and cell motility [179].

### 4.3. Cross-Linking and Bundling Proteins Interact with CSCs

Fascin, as an actin-binding protein, directly mediates chemoresistance in breast cancer by activating FAK [180]. Moreover, fascin activates β-catenin signaling and promotes breast CSC function, mainly through focal adhesion kinase (FAK) [181]. The upregulation of fascin expression results in cytoskeletal changes that promote metastasis [182]. After the knockdown of fascin in ovarian cancer stromal cells with high fascin expression, we found that CSC activity, metastasis, and the EMT were reduced through pathways such as Rac1, RhoA, and NF-κB [183]. As a result, the increased expression of fascin in most aggressive cancers often represents the possibility of metastasis. FLNA is able to remodel the actin cytoskeleton of CSCs, leading to enhanced tumor metastasis [184]. It interacts with Rho GTPases, which activate cell migration, and Ras GTPases, which inhibit cell migration, to promote metastasis [184]. The downregulation of FLNA increases the destruction of single- and double-stranded DNA in tumor cells after cisplatin therapy, increasing chemosensitivity [185]. Moreover, a lack of FLNA arrests the cell cycle in the G2/M phase and increases angiogenesis by promoting the expression of VEGF [186]. In head and neck CSCs, the activation of CD44 alters FLN expression, resulting in enhanced cell migration and chemoresistance [187]. According to research, spectrins may be closely associated with tumorigenesis, progression and metastatic processes [188]. Spectrin is highly expressed in early-stage colorectal cancer but lower in advanced or metastatic cells [189]. It was shown that colorectal cancer cell viability and cell contacts were reduced and metastasis was increased when spectrin was knocked out. Furthermore, a marked decrease in spectrin expression may result in the loss of DNA mismatch repair proteins [190]. In addition, β2 spectrin was shown to inhibit the properties of hepatic CSCs through β-catenin-induced differentiation, which is a new strategy for hepatocellular carcinoma prevention and differentiation therapy [191]. ACTN is involved in cell differentiation and cancer metastasis by modulating the activity of several signaling pathways and recombinant actin filaments [93]. It was shown that the potential mechanism of the ACTN4-mediated properties of CSCs mainly involves the Akt/GSK-3β/β-catenin axis. The ACTN4-mediated stabilization of β-catenin is closely related to Akt/GSK-3β signaling. ACTN4 promotes the EMT and cell cycle progression by stabilizing β-catenin, maintaining the properties of CSCs, and leading to drug resistance [192,193]. Furthermore, studies have shown that high levels of ACTN4 expression are related to malignancy, metastasis, poor prognosis, and chemotherapy resistance in numerous tumors, including pancreatic, ovarian, and bladder cancers [93,194].

### 4.4. Anchoring Proteins and CSCs

The increased expression of ezrin leads to increased malignancy and decreased survival in aggressive cancers [195]. Ezrin tends to be expressed more on the apical surface of tissues in non-invasive tumors, whereas in invasive cell lines, it tends to be expressed in local membrane folds and filopodia, which are more favorable for promoting metastasis [196]. Furthermore, the ectopic expression of phosphomimetic forms of ezrin promotes cancer progression and metastasis in vitro and in vivo [197]. Ezrin and CD44 are co-highly expressed in breast CSCs, which is associated not only with poor prognosis but also with the resistance of CSCs to chemotherapy [198]. In pancreatic ductal adenocarcinoma (PDAC), the level of ezrin in CSCs is significantly higher than that in normal cancer cells. The severe impairment of CSC frequency, self-renewal capacity, and tumor initiation potential was observed following the knockout or inhibition of ezrin. These all suggested that ezrin affects the properties of CSCs in PDAC [199]. Ezrin is associated with defective adhesion turnover and a loss of directional migration, leading to tumor invasion and metastasis [200]. The potential mechanism could be as follows. On the one hand, ezrin connects the cytoplasmic tail of CD44 to F-actin, leading to cytoskeletal remodeling, and changes in actin cytoskeletal dynamics and cell shape could guide stem cell differentiation [201]. On the other hand, ezrin can also maintain CSC properties by regulating actin polymerization through ROCK inhibition [199]. Moesin, like other ERM proteins, has also been implicated in cancer progression [202]. Moesin is commonly overexpressed in high-grade glioblastoma, and its mode of action correlates with the CSC marker CD44. The main mechanism of action of moesin is to increase the expression of CD44 in the Wnt/β-catenin signaling pathway and to enhance the positive feedback effect on this pathway. Furthermore, moesin was shown to increase the expression of SOX2, promoting the functional transition of glioblastoma to an aggressive stem cell phenotype [203]. Merlin is encoded by the tumor suppressor gene NF2 [204]. This protein regulates YAP/TAZ proteins through the merlin/NF2/YAP/TAZ axis [205]. YAP/TAZ proteins are key regulators of the properties of breast cancer CSCs [206].

### 4.5. Capping and Severing Proteins Interact with CSCs

Gelsolin is closely associated with properties such as oncogenic phenotype, the EMT, cell motility, apoptosis, proliferation and differentiation [207]. Furthermore, gelsolin interferes with TGF-β1-driven CSC differentiation through the EMT process in breast cancer cells [208]. Gelsolin affects the differentiation and properties of stem cells by regulating stem cell-related transcription factors such as Nanog, SOX2, and OCT4 [208]. Chemotherapeutic drugs induce hepatocellular carcinoma cell death by activating cofilin-1, a process associated with the interaction of Bcl-2-associated X protein and ROS accumulation. Thus, the phosphorylation of cofilin-1 leads to chemoresistance [209]. High levels of cofilin-1 have been shown to be prognostic biomarkers and predictors of drug resistance [93]. The overexpression of cofilin in prostate cancer leads to an enhanced EMT and promotes metastasis and CSC properties [210]. Studies have reported that the knockdown of villin in specific cell lines using siRNA resulted in cell growth arrest, demonstrating its importance in carcinogenesis [211]. Villin can also be used as a marker of gastric CSCs and a biomarker of metastatic lung adenocarcinoma [212,213].

### 4.6. Stabilizing and Signaling Proteins Interact with CSCs

Highly expressed tropomodulins in hepatocellular carcinoma lead to increased invasiveness, metastasis, CSC properties, and matrix metalloproteinase (MMP) expression through the activation of the PI3K–AKT signaling pathway [214]. Tropomodulins increase the expression of MMP-13 and NF-κB in breast cancer, which contributes to enhanced tumor invasion, stemness and metastasis [215]. VASP is involved in ECM-mediated β1-integrin-FAK–YAP/TAZ signaling, which is closely related to the regulation of CSC properties [216]. Furthermore, the increased expression of Ena/VASP in PDAC and colorectal cancer (CRC) were found to be significantly associated with liver metastasis and lower survival [216]. In gastric cancer cells, the expression of VASP can be inhibited by miR-4455, thereby reducing VASP-mediated properties such as proliferation, migration, stemness and invasion [217].

### 4.7. Microtubule-Associated Proteins Interact with CSCs

Tubulin regulates the EMT and contributes to the formation of lamellar and filopodia, promoting cancer cell stemness and metastasis [218]. The ectopic expression of Snail or Twist facilitates α-tubulin decarboxylation and microtubulin-based microtentacle formation, which aid in invasion and migration [219]. Tau can modulate cell cycle processes and related signaling pathways in cancer to affect stem cell-like phenotypes [93]. For example, Tau activates the MAPK pathway involved in prostate cancer progression by binding to PI3K [220]. The high expression of tau mRNA in breast cancer often indicates chemoresistance [221]. It was confirmed that katanin contributes to the formation of CSCs, leading to metastasis [222]. The main principle may be that katanin acts as a microtubule-severing protein that cleaves cellular microtubules into short pieces and activates JNK [223]. In addition, upregulated katanin may increase microtubule dynamics, accelerate the cell cycle, and increase cell viability and cell migration, thereby promoting tumor metastasis [224]. A previous study suggested the involvement of microtubule stabilizer ATIP3 in the inhibition of ERK1/2 activity. ATIP3 leads to the inhibition of CSCs and the EMT through ATIP3/ERK1/2-Snai2 signaling, reducing cell proliferation, migration and invasion [225]. Among kinesins, KIF11 was found to enhance the stemness of cancer cells by promoting the expression of stemness transcription factors (NANOG and OCT4), leading to cell proliferation and resistance to chemotherapeutic agents [226]. STMN1 leads to microtubule depolymerization, which promotes the activation of Rho, thereby enhancing the EMT and cell stemness [227]. Moreover, microtubule disruption promotes the assembly of adherent spots and enhances cell migration [228]. CAMSAP3 protects lung cancer cells from the EMT by inhibiting Akt activity through microtubule regulation, whereas CAMSAP3 deficiency promotes the EMT and stemness maintenance in these cells [229].

### 4.8. Other Components of the Cytoskeleton on CSCs

The nuclear transfer of cells through the dense extracellular matrix is one of the most important steps in the process of cancer metastasis [230]. Thus, nuclear translocation is considered to be a key limiting factor for the efficient spatial migration of cancer cells [230]. It was demonstrated that myosin IIB enhances the ability of nuclear translocation in breast CSCs, thereby enhancing stem cell invasiveness [231]. Myosin IIB combines a nuclear scaffold structure with the actin cytoskeleton to facilitate the extrusion of nuclei through narrow spaces, resulting in effective 3D collagen invasion [232]. In addition, myosin IIA is involved in promoting the EMT, and the transition between myosin IIB and myosin IIC is critical for the EMT, contributing to stemness maintenance by influencing cell contractility [233,234]. Vimentin, a type III IF, is one of the key biomarkers for the EMT and is usually upregulated during cancer metastasis [235]. Vimentin regulates EMT-related genes, including Twist, Snail, ZEB1/2, and Slug, as well as key epigenetic factors [236]. Moreover, it relies on inducing genes associated with self-renewal to inhibit cell differentiation and to upregulate their pluripotent potential, thereby increasing the stemness of CSCs and promoting tumor metastasis and chemoresistance [237]. Nestin is closely related to self-renewal capacity and is considered a stem marker for neurogenic tumors and epithelial or mesenchymal tumors [238]. Nestin may be a useful biomarker and a new target for inhibiting tumor angiogenesis due to its more widespread expression in the proliferating vessels of PDAC [239]. High levels of nestin expression in breast cancer patients are correlated with the upregulation of VEGF, cancer stem cell markers, and proteins that activate Wnt/β-catenin to initiate proliferation [240]. Several keratins (KRT6, 14, 16, and 17) have been reported to be involved in the regulation of different types of cancer stem cells [241]. The interkeratin fusion between KRT6 and KRT14 promotes CSC-related properties in oral squamous cell carcinoma [241]. KRT16 can promote cancer drug resistance and stemness by interacting with the β5-integrin/c-Met signaling pathway [242]. KRT17 regulates stemness and chemoresistance by binding to β4-integrin/FAK, Src, or β-catenin [243] (Table 1).

### 4.9. Mitochondria-Cytoskeleton Interactions and CSCs

CSCs exhibit elevated mitochondrial fusion, and their metabolism relies on a rearranged cytoskeletal network and OXPHOS [12]. Increased mitochondrial fusion encourages ATP synthesis by OXPHOS, addressing the energy limitation problem for CSC survival [244]. In addition to functioning as a crucial metabolic enzyme in glycolysis, aldolase also interacts with cytoskeletal elements that regulate actin polymerization [245]. Through cytoskeletal rearrangements leading to the spatial redistribution of aldolase, PI3K plays an AKT-independent role in altering glycolysis, thereby increasing energy metabolism [12]. Cytoskeletal rearrangements or regulatory mechanisms between cellular bioenergetics and cytoskeletal regulators are critical for understanding the responses of cancer cells, especially CSCs, to different stimuli [12]. The EMT program has been identified as one of the key regulators of the CSC phenotype [45]. The EMT is also regulated by cytoskeleton–mitochondrial interactions [12]. The EMT is determined in part by the morphological reprogramming of cellular architecture and sustained by a reconstituted cytoskeleton [246]. The aggregation of mitochondria near the cell membrane is essential to facilitate the formation of cytomotor structures such as pseudopods during the EMT [247]. Studies have suggested that ROS may participate in the regulation of the EMT through actin reorganization [12,248].

## 5. Therapeutic Strategies Targeting Cytoskeleton

### 5.1. Therapeutic Strategies Targeting Actin

The cytoskeleton is essential for the invasion and migration of cancer cells, making it a promising therapeutic target. Although the concept of cancer therapy targeting actin is not new, other healthy cells may be affected because of severe off-target effects [249]. Therefore, the issue of the clinical application of actin-targeted therapy remains a pressing challenge. At present, a variety of actin toxins and inhibitors, including phalloidin, cytochalasins, jasplakinolide, latrunculins, wiskostatin, CK-666, and CK-869, are widely used for research [250,251,252]. However, with severe and widespread cytotoxicity, the clinical application of these drugs has not been implemented. Thus, it is crucial to develop actin inhibitors with strong specificity and high safety to treat cancer cells. Considering the specificity of actin, researchers have focused on actin-nucleating agents. The use of small-molecule-targeted formalin has been shown to be potentially beneficial for cancer treatment. The small-molecule inhibitor SMIFH2 inhibits formalin activity, and SMIFH2 binds to the FH2 structural domain of actin nucleation, inhibiting actin nucleation and elongation [253]. The use of SMIFH2 was found to enhance the sensitivity of ovarian cancer cells to cisplatin or paclitaxel [254]. Rho expression tends to be increased in tumors, and actin dynamic function in cancer cells correlates with Rho activity [255]. Therefore, the inhibition of Rho or upstream signaling regulators of Rho can block abnormal cytoskeletal activity and may be a promising strategy for cancer therapy [256]. Cdc42 is a small GTPase that activates a variety of downstream effector molecules, including actin-related proteins, kinases, and phospholipases [257]. The Food and Drug Administration (FDA)-approved analgesic drug R-ketorolac (Toradol) inhibits Cdc42 and Rac1 in ovarian cancer cells and is currently in clinical trials (NCT02470299) [258]. Another effective drug, MBQ-167, is an inhibitor of Rac/Cdc42 both in vivo and in vitro [259]. It has been shown to suppress the motility viability and clumping of breast cancer cells, but further optimization and development are required before clinical trials [256]. Rac is often overexpressed or overactivated in a variety of cancers and is also a small GTPase protein [260]. The drugs currently being developed to inhibit Rac activity have only been tested at the cancer cell level, but none appear to be entering clinical trials [259]. ROCK is another target proposed in multiple studies [256]. ROCK1/2 serine/threonine kinases modulate cell morphology, as well as actin cytoskeleton reorganization, by phosphorylating various ABPs, such as ERM proteins [261]. The inhibition of ROCK1/2 could theoretically disrupt the cytoskeletal dynamics of cancer cell actin and provide therapeutic benefits [262]. However, another study suggested that ROCK inhibition may activate an alternative pathway leading to a more aggressive migratory phenotype [263]. Additionally, currently used ROCK inhibitors lack selectivity for ROCK1 and ROCK2, which have distinct roles in the regulation of cytoskeletal networks [264]. The use of non-selective inhibitors may actually have a promoting effect on certain malignancies and the tumor microenvironment [265]. The combination of novel selective ROCK inhibitors with different anticancer drugs for cancer treatment is anticipated and exciting.

### 5.2. Therapeutic Strategies Targeting ABPs

Profilin has the potential to be a therapeutic target against a variety of cancers because of its role in cytoskeletal regulation and its location in cancer signaling cascades [266]. Small molecule screens have identified two small molecules (C1 and C2) that prevent profilin from interacting with actin monomers [267]. However, its mechanism and safety need to be further studied before clinical trials. Tβ4 also has potential therapeutic effects on cancer. Silencing the Tβ4 gene in non-small cell lung cancer was found to inhibit tumor progression, suggesting that Tβ4 could be a candidate target for therapy [268]. Tβ4 tends to be highly expressed in rectal CSCs. Interestingly, when using lentivirus to reduce Tβ4 levels in rectal cancer stem cells, this treatment significantly reduced tumor size and aggressiveness in mice [172]. A few newly synthesized drugs have been detected to act as active fascin inhibitors to treat cancer [269]. Compound G2 was found to inhibit fascin-1-directed actin remodeling, an action that caused the destruction of filamentous pseudopods and minimized the migratory and invasive characteristics of colorectal cancer cells both in vitro and in vivo [270]. However, the side effects of this treatment are not fully understood and need to be further studied. Additionally, compounds 3 and 14 were found to substantially downregulate fascin-1 and abolish the EMT, leading to a reduction in the invasiveness and metastatic ability of cancer cells [271]. However, further research is needed to study how these compounds work in vivo and to address any side effects as much as possible. Among them, the small-molecule fascin inhibitor NP-G2-044 has been shown to block tumor invasion and metastasis. This orally available inhibitor binds to fascin and blocks the interaction of fascin with actin filaments [272]. A multicenter Phase 1A clinical trial was designed to evaluate the safety and tolerability of NP-G2-044 in a single daily oral dose for the treatment of patients with refractory solid tumor malignancies (NCT03199586). Overall, the results showed the good absorption and distribution of NP-G2-044 in humans, with initial signals of antitumor and antimetastatic activity observed and no drug-related serious adverse events, dose-limiting toxicities, or patient deaths [273]. Raltegravir is a human immunodeficiency virus 1 integrase inhibitor that disrupts cell motility by directly acting on fascin-1 to cause the breakdown of the actin cytoskeleton [274]. However, its safety and efficacy in humans remain to be tested. Salinomycin was identified as an inhibitor of fascin-1, an ion carrier and antibiotic in its own right. Salinomycin relocates fascin-1 from filamentous pseudopods in PDAC cells, disrupting actin cytoskeleton remodeling and inhibiting cancer metastasis to secondary sites [275]. The antidepressant imipramine has also been identified as a novel fascin-1 inhibitor that significantly reduces fascin-1 expression and disrupts filamentous pseudopod formation and cytoskeletal remodeling [276]. Clinical trials on imipramine are currently underway in ER+/triple-negative breast cancer (NCT03122444) and recurrent glioblastoma (NCT04863950) [269].

The inhibition of ezrin phosphorylation may be an effective strategy for cancer treatment. Researchers screened a small-molecule library of multiple compounds that might interact with ezrin by ion resonance (SPR) technology, and two of these compounds—NSC668394 and NSC305787—were found to have a strong binding affinity for ezrin [277]. They can significantly inhibit the phosphorylation of ezrin, inhibit the interaction between ezrin and F-actin, and achieve the inhibition of oncogenic activities including osteosarcoma cell invasion, migration, and lung metastasis [278]. Furthermore, a study found that NSC668394 in combination with lapatinib, a drug targeting HER2 and EGFR, enhanced the induction of apoptosis and the inhibition of breast cancer cell proliferation [279]. Ezrin is essential for cancer progression, acting as a scaffolding protein and interacting with related proteins in cancer cells [197]. Hence, designing inhibitors to interfere with the interaction of ezrin with related proteins may be another strategy for cancer therapy. For example, small molecules that interfere with the ezrin–L1CAM interaction may be promising therapeutic agents for colorectal cancer [280]. Furthermore, the treatment of ERMs with cytochalasin B was shown to remarkably suppress the metastasis and phagocytic activity of melanoma cells, indicating that the inhibition of actin assembly by ezrin inhibitors may be a potential therapeutic tool for melanoma [281]. Another study found that G1749-A1771 siRNA targeting ezrin mRNA effectively downregulated the expression of ezrin, contributing to the induction of apoptosis and the inhibition of cell proliferation in osteosarcoma cells [282]. In addition, AKT inhibitors (MK2206) or PI3K inhibitors (LY294002) can block ezrin-mediated tumor growth and metastasis by inhibiting the PI3K/AKT signaling pathway [197]. The multi-kinase inhibitor sorafenib (BAY43-9006) promoted apoptosis by inhibiting the ezrin pathway and inhibited angiogenesis and metastasis in a mouse model of osteosarcoma [283]. There are also many cancer drugs targeting ezrin in natural compounds. Recent studies have shown that baicalin exerts antitumor effects by inducing apoptosis and inhibiting cell proliferation and invasion by inhibiting the expression of ezrin [284]. In addition, the binding of celastrol to ROCK2 inhibits the migration of hepatocellular carcinoma, mainly through the impaired ROCK2-mediated phosphorylation of ezrin, resulting in ineffective ezrin activation [285].

### 5.3. Therapeutic Strategies Targeting Microtubules and IFs

Considering the important role of microtubules in the cytoskeleton, drugs targeting microtubule dynamics are one of the most effective treatments [286]. For example, one of the first compounds to target the cytoskeleton to treat cancer was paclitaxel (PTX), which stabilizes microtubules and effectively prevents cell division in a wide range of cancers, including lung, ovarian, and breast cancers [287]. However, the effectiveness of PTX is limited by various side effects. The main side effects of PTX are allergy and neuropathy. PTX hypersensitivity reactions are usually seen within the first ten minutes of administration and include dyspnea, bronchospasm, urticaria, abdominal pain, fever, or chills, which usually result in the immediate discontinuation of therapy [288]. In addition, the cardiotoxicity caused by PTX administration cannot be ignored. Effective inhibitors of microtubule dynamics also include periwinkle alkaloids, which is widely used in cancer therapy [12]. Given the importance of intermediate filament proteins in various tumor activities, treatment targeting intermediate filaments and their associated signaling networks may also be a promising therapeutic strategy [289]. The naturally derived bioactive compound withaferin-A targets and induces vimentin cleavage and inhibits tumor progression and metastasis in mouse models [290]. Although withaferin-A is currently the only small molecule that inhibits the structure and function of intermediate filaments, it also acts on several other cellular components and lacks specificity [291]. Therefore, there is an urgent need to develop inhibitors that exclusively target intermediate filament proteins to modulate their function, which will be very important for the clinical treatment of cancer patients.

### 5.4. Therapeutic Strategies Targeting MAPs

The expression of MAPs can greatly influence the efficacy of microtubule-targeted therapy [292]. The MAP tau was identified as a predictive marker for a pathological complete response to paclitaxel in breast cancer patients. Low levels of tau protein expression make mitosis and cytoskeletal microtubules more sensitive to paclitaxel disruption [293]. After long-term treatment, drug resistance has become a major problem in targeting microtubule therapy. These resistance mechanisms are associated with alterations in the microtubule proteins themselves, including alterations in microtubule protein isoform expression, post-translational modifications of microtubule proteins, and the acquisition of microtubule protein mutations [294]. The purine-type compound 5a affects the structure of microtubules and causes apoptosis in cancer cells by targeting the microtubule cleavage protein katanin. This pharmacological effect may bypass the primary resistance mechanism described above. Thus, 5a and its analogs may be new therapeutic options for targeting katanin [223]. KIF11 is currently the most well-studied kinesin in the clinical setting. Ispinesib, a quinazolinone derivative, is the first KIF11 inhibitor to be studied in clinical trials [295]. In a phase I trial (NCT00089973) evaluating the safety and efficacy of ispinesib in breast cancer, antitumor activity was detected in 20% of patients and stable disease was noted in 73% of patients, with 27% having stable disease for 90 days or longer. The most common adverse events reported were neutropenia, elevated liver transaminases, and diarrhea [296]. SB743921 is a recently discovered inhibitor of KIF11. In a clinical trial (NCT00136513) of SP743921 in patients with advanced solid tumors, the drug showed encouraging efficacy without serious toxicity [297]. Other KIF11 inhibitors that have shown promise in cancer therapy include curcumin and various tetrahydro-β-carboline-acetonide hybrids and thione derivatives. Curcumin is a non-specific plant polyphenol extracted from turmeric that exhibits antioxidant, anti-inflammatory, antibacterial and antiviral biological activities [298]. Based on extensive studies, stathmin has recently emerged as a promising drug candidate for the treatment of solid malignancies. To reduce stathmin transcripts in vitro and in vivo and to explore therapeutic approaches against stathmin, a range of specific anti-stathmin agents are being developed [299]. Anti-stathmin nuclease gene delivery via adenovirus reduces multiplication and clonality in breast cancer cells with and without estrogen receptors [300]. The stathmin promoter-driven Aurora-A shRNA adenoviral pathway can be used to manage breast cancer as a complementary tumor-specific therapy [301].

### 5.5. Therapeutic Strategies Targeting the Metabolism of CSCs

Mitochondrial fusion is important for the energy metabolism of CSCs, so the inhibition of mitochondrial fusion may be a candidate cancer therapeutic strategy. Changes in mitochondrial fusion proteins affect mitochondrial morphology and integrity, making these proteins ideal therapeutic targets [302]. β II protein kinase C (βIIPKC), a selective inhibitor of mitochondrial fusion proteins, phosphorylates fusion proteins and causes the partial loss of GTPase activity, resulting in the fragmentation and dysfunction of mitochondria [303]. OXPHOS inhibitors are expected to be used for the targeted therapy of OXPHOS-dependent tumors. Metformin is one of the most promising inhibitors of OXPHOS, a diabetes agent for cancer therapy [304]. Benzformin is an alternative to metformin that has the advantage of a greater affinity for mitochondrial membranes and easier transport in cancer cells [305]. A novel metformin derivative (IM156) primarily acts on slowly circulating tumor cells that have the ability to evade conventional chemotherapy [306]. These therapies have been shown to inhibit mitochondrial function and CSC survival, but consideration ought to be given to whether the suppression of OXPHOS or a specific component induces an alternative pathway that affects ultimate anticancer efficacy (Table 2).

## 6. Conclusions

In this review, we summarize the mechanisms by which interactions between the cytoskeleton, cytoskeleton-associated proteins and CSCs lead to tumor metastasis and drug resistance. The effects of various cytoskeletal components, including ABPs and MAPs, and cytoskeletal reorganization on CSCs are emphatically expounded. Additionally, the main metabolic mode of CSCs, OXPHOS, is also modulated by cytoskeletal–mitochondrial interactions. Thus, a detailed understanding of the interactions between the CSCs and cytoskeleton facilitates the development of new cancer treatment strategies to provide better therapy for metastatic and drug-resistant patients.

## Figures and Tables

**Table 1:** Mechanism of cytoskeleton and cytoskeleton-related proteins on CSCs.

Class	Proteins	Mechanism	Effect
	Actin	Activation of the downstream Rho/ROCK pathway via YAP/TAZ	Facilitating the survival of CSCs
Monomer-binding proteins	Profilin	Direct regulation of stem cell-associated transcription factors	Maintaining the stemness of CSCs
	Thymosin β4	Activation of the BMP pathway, followed by JNK activation via the TAB1 and TAK1 complexes	Maintaining the stemness of CSCs
	Twinfilin	Enhancing the activity of the MKL1 and actin cytoskeleton dynamics	Facilitating the survival of CSCs
Cross-linking and bundling proteins	Fascin	Activation of β-catenin protein signaling via FAK	Promoting CSC function
	Filaminin	Interacts with Rho GTPases that activate cell migration and Ras GTPases that inhibit cell migration	Promoting CSC function
	Spectrin	Inhibition of CSCs by β-catenin-induced differentiation	Inhibiting the properties of CSCs
	α actinin	Acts through the Akt/GSK-3β/β-catenin axis	Maintaining the stemness of CSCs
Anchoring proteins	Ezrin	Regulation of actin polymerization by ROCK inhibition	Maintaining the properties of CSCs
	Moesin	Enhancement of positive feedback on the Wnt/β-catenin signaling pathway by increasing the expression of CD44	Promoting CSC function
	Merlin	Adjusting the YAP/TAZ pathway via the merlin/NF2/YAP/TAZ axis	Promoting CSC function
Capping and severing proteins	Gelsolin	Direct regulation of stem cell-associated transcription factors	Maintaining the properties of CSCs
	Cofilin	Acts by promoting EMT expression	Maintaining the properties of CSCs
Stabilizing proteins	Tropomodulin	Increased expression of MMP-13 and NF-κB and the activation of the PI3K–AKT signaling pathway	Promoting CSC function
Signaling proteins	ENA/VASP	ECM-mediated β1-integrin-FAK–YAP/TAZ signaling pathway	Maintaining the properties of CSCs
	Tubulin	Regulating EMT and contributing to the formation of lamellar filopodia	Promoting CSC function
Microtubule lattice-binding proteins	Tau	Activating the MAPK pathway by binding to PI3K	Maintaining the properties of CSCs
	katanin	Activation of JNK by cutting cell microtubules into short segments	Promoting CSC function
	ATIP3	Inhibition through ATIP3/ERK1/2-Snai2 signaling	Inhibiting the properties of CSCs
Microtubule motor proteins	Kinesins	Promoting the expression of stem transcription factors (NANOG and OCT4)	Maintaining the properties of CSCs
Multiple site microtubule-binding proteins	STMN1	Activates Rho by promoting microtubule depolymerization	Promoting CSC function
Microtubule minus-end-binding proteins	CAMSAPs	Inhibition of Akt activity through microtubule regulation	Inhibiting the properties of CSCs
	Myosin	Involved in promoting the EMT and enhancing the nuclear translocation of CSC	Maintaining the properties of CSCs
Intermediate filaments	Vimentin	Regulation of EMT-related genes, including Twist, Snail, ZEB1/2 and Slug	Maintaining the properties of CSCs
	Nestin	Upregulation of VEGF, cancer stem cell markers, and proteins that activate Wnt/β-catenin to initiate proliferation	Promoting CSC function
	Keratins	Interacting with the β5-integrin/c-Met signaling pathway	Promoting CSC function
		Binding to β4-integrin/FAK or Src or β-catenin	

**Table 2:** Overview of clinical trials and experiments targeting the cytoskeleton.

Categories	Drug Name	Mechanism	Clinical Trial	NCT Registry Number/Ref.
Actin	SMIFH2	Inhibiting actin nucleation and elongation	Experimental	[254]
	Toradol	GTPase inhibition	Active, not recruiting	NCT02470299
	MBQ-167	Rac/Cdc42 inhibitor	Experimental	[256]
Profilin	C1 and C2	Preventing profilin from interacting with actin monomers	Experimental	[267]
Tβ4	Tβ4 inhibitors	Silencing the Tβ4 gene	Experimental	[172,268]
Fascin	NP-G2-044	Inhibiting fascin-1-directed actin remodeling	Completed	NCT03199586
	Compounds 3 and 14	Downregulating fascin-1	Experimental	[271]
	Raltegravir	Inhibitor of human immunodeficiency virus 1 integrase	Experimental	[274]
	Salinomycin	Fascin-1 inhibition	Experimental	[275]
	Imipramine	Fascin-1 inhibition	Early Phase 1	NCT03122444
			II	NCT04863950
Ezrin	NSC305787	Inhibiting the phosphorylation of ezrin	Experimental	[278]
	NSC668394		Experimental	[279]
	Cytochalasin B	Inhibition of actin assembly	Experimental	[281]
	LY294002	PI3K inhibitor	Experimental	[197]
	MK2206	AKT inhibitor		
	BAY43-9006	Multi-kinase inhibitor	Experimental	[283]
	Baicalin	Inhibitor of ezrin	Experimental	[284]
	Celastrol	Impairing the phosphorylation of ezrin	Experimental	[285]
Microtubules	Paclitaxel	Stabilizing microtubules	Clinical medication	[287]
	Taxanes	Inhibitors of microtubule dynamics	Clinical medication	[12]
	Vinca alkaloids			
Vimentin	Withaferin-A	Inducing vimentin cleavage	Experimental	[290]
Kinesin	Purine compound 5a	Regulating katanin’s cut-off activities	Experimental	[223]
	Ispinesib	A kinesin spindle protein inhibitor	Completed	NCT00089973
	SB-743921			NCT00136513
Stathmin	Anti-stathmin adenovirus	Cell cycle inhibition	Experimental	[300]
	Aurora A shRNA	Inhibitor of stathmin	Experimental	[301]
Mitochondria	βIIPKC	Inhibitor of mitochondrial fusion proteins	Experimental	[303]
	Metformin	Inhibitors of OXPHOS	Experimental	[304]
	Benzformin		Experimental	[305]
	IM156	Metformin derivative	Experimental	[306]

## Data Availability

Not applicable.
